# Physical Activity and Health Perception in Aging: Do Body Mass and Satisfaction Matter? A Three-Path Mediated Link

**DOI:** 10.1371/journal.pone.0160805

**Published:** 2016-09-09

**Authors:** Giancarlo Condello, Laura Capranica, Joel Stager, Roberta Forte, Simone Falbo, Angela Di Baldassarre, Cristina Segura-Garcia, Caterina Pesce

**Affiliations:** 1 Department of Movement, Human and Health Sciences, University of Rome Foro Italico, Rome, Italy; 2 Department of Kinesiology, Indiana University, Bloomington, IN, United States of America; 3 Department of Medicine and Aging Sciences, "G. d'Annunzio" University of Chieti-Pescara, Chieti, Italy; 4 Department of Health Sciences, University Magna Graecia, Catanzaro, Italy; Universidad Pablo de Olavide, SPAIN

## Abstract

Although ageing people could benefit from healthy diet and physical activity to maintain health and quality of life, further understandings of the diet- and physical activity-related mechanisms that may cause changes in health and quality of life perception are necessary. The purpose of the study was to investigate the effect of eating attitudes, body mass and image satisfaction, and exercise dependence in the relationship between physical activity and health and quality of life perception in older individuals. Hundred and seventy-nine late middle-aged, (55–64 yrs), young-old (65–74 yrs), and old (75–84 yrs) senior athletes (n = 56), physically active (n = 58) or sedentary adults (n = 65) were submitted to anthropometric evaluations (body mass, height) and self-reported questionnaires: Body Image Dimensional Assessment, Exercise Dependence Scale, Eating Attitude Test, and Short Form Health Survey (Physical Component Summary [PCS] and Mental Component Summary [MCS] of and health and quality of life perception). Senior athletes, physically active, and sedentary participants subgroups differed (P<0.05) from each other in body mass index (BMI) and several components of body image and exercise dependence. Senior athletes showed, compared to their sedentary counterparts, further differences (P<0.05) in eating attitudes and in both PCS and MCS. Mediation analysis showed that the relationship between physical activity habit and MCS, but not PCS, was indirectly explained by a serial mediation chain composed of objective BMI and subjective body image (dis)satisfaction. Findings confirm the relevant role of physically active life habits for older individuals to perceive good physical and mental health. The novelty of the three-path mediated link between physical activity level and mental health perception suggests that the beneficial effect of a physically active lifestyle on weight control can positively impinge on the cognitive-emotional dimension of mental health by ensuring the maintenance, also at older age, of a satisfactory body image.

## Introduction

In light of the increasing life expectancy of western citizens, several organizations and government agencies have endorsed the benefits of a healthy lifestyle and healthy diet [1,2] In fact, the combination of insufficient physical activity and high-energy intakes is responsible for the actual high incidence of overweight and obesity, which is linked to several diseases [3]. In the health sector, awareness has increased on the role played by active lifestyles for successful aging and a worldwide consensus supports the need for lifelong physical activity [2]. Conversely, aging individuals tend to be sedentary [4,5] and to adopt unhealthy eating attitudes [6].

Aging is a multi-factorial process leading to physical, psychological, and health declines associated with several chronic conditions, although impairment of health status and well-being varies considerably between individuals [7]. In the last decades, there has been a shift in health promotion policies from the goal of increasing the span of healthy life to that of improving overall quality of life and well-being [8,9]. This shift of the focus toward subjectively perceived health-related quality of life is relevant in the light of evidence showing that even if physical health and function become poorer with aging, quality of life perception can remain high. This is explained by the multifaceted nature of perceived health-related quality of life and the relevance of its mental health component [10,11].

Aging does not necessarily worsen quality of life when controlling for other commonly age-related influences [12]. According to representative epidemiological surveys from different countries [10,11,13–16], main determinants of health-related quality of life in older adulthood range from individual-level factors as overall health, presence/absence of functionally limiting disease, self-esteem, and cognitive efficiency/dysfunction, to socio-environmental factors as the presence/lack of trusting social relationships and financial resources/hardships.

Among the lifestyle factors that can counteract the decline of quality of life with advancing age, physical activity, and diet have been claimed to play a pivotal role [8,13,17–19]. The strong influence of physical activity on health-related quality of life perception is explained by the fact that it positively impacts not only physical health and function, thus lowering the incidence of non-communicable diseases [20], but also emotional and cognitive components of mental health [21,22].

Physical activity may have different meanings, broadly ranging from unstructured activities integrated into everyday life, exercise performed in a prearranged, deliberate, and repetitive manner, to competitive sport [23]. In particular, senior athletes who keep training and competing over 50 years of age are considered an example of successful aging [7]. In fact, they tend to preserve lean body mass, healthy weight, and high levels of fitness and physical tasks of daily life, which significantly reduce their risk of diseases [24,25].

For older athletes, participation in competitive sport represents also an important strategy to express youthfulness and negotiate the remarkable bodily changes associated with advancing years [26]. Concerns about appearance could diminish with advancing years [27] when health and physical capability become more imperative and weight gain is considered as part of the inevitable aging process. On the other side, body image dissatisfaction could emerge with aging and determine negative attitudes [28]. Body image is a multidimensional and complex construct, which includes cognitive, perceptual, emotional, and behavioural aspects [29]. Body image varies along the aging process especially in women [30] with relations to weight control, eating disturbances, and depression, all of which can negatively impact health-related quality of life [31,32]. Overweight and obese adults with body image misconceptions could be less prone to adopt weight-control behaviours [33], whereas exercisers seem to have a more positive body image than nonexercisers [34].

Although active lifestyles, positive eating attitudes and health could be associated with a functional (“want to”) commitment to sport, senior athletes might show an obligatory (“have to”) commitment to training [35], which could determine addictive tendencies toward exercise dependence [36]. Furthermore, to maintain or improve their performance, senior athletes could adopt radical dietary habits [37], which might affect their perception of health-related quality of life.

In sum, there is strong scientific evidence and policymakers’ awareness of the relationship linking diet and physical activity to quality of life in older adulthood [8,13,17–19]. However, successful transitioning into evidence informed policy and practice, design and implementation of physical activity programs for older adults requires the understanding of mechanisms that underlie such linkage [19].

At present, the above described piecemeal relationships between older adults’ engagement in sport and exercise programmes, eating attitudes, perceived body image, and health-related quality of life perception allow to infer, but do not provide compelling evidence on the potential mechanisms that explain why behavioural nutrition and activity habits contribute to the perception of having a good quality of life. The present study was aimed at addressing this issue.

The first objective was to evaluate if the type and degree of physical activity commitment (i.e., being senior athletes, physically active or sedentary older individuals) is associated with different diet-related and physical activity-related personal characteristics and behavioural attitudes. The second and main objective was to evaluate if such characteristics and attitudes can explain why physical activity habits affect health and quality of life perception.

Based on the evidence that physical activity affects body weight and image and body dissatisfaction has linkages to eating disorders and exercise dependence [3,34,38,39], we hypothesized that eating attitudes, body weight and image, and exercise dependence may belong to a mediational chain through which habitual participation to sport and physical activity in old adulthood contribute to the subjective perception of physical and mental health and quality of life.

## Materials and Methods

### Participants

The Ethics Committee Azienda Policlinico Umberto I (Rome, Italy, reference number: Prot. 451/13) approved the study and all participants provided written informed consent for participation and publication. Participating were free of opting out at any time (i.e., not completing all the evaluations) without providing any reason. Participants were recruited according to the following eligibility criteria: 1) age between 55 and 84 yrs; and 2) not self-reported diagnosis of psychiatric or somatic illnesses. A stratified sampled for the declared physical activity level was applied. Accordingly, there were three types of eligible participants: athletes engaged in competitive running or swimming (≥3 training sessions^.^week^-1^) at national or international levels; physically active individuals engaged in regular structured physical activity programmes (≥2 session^.^week^-1^); and sedentary individuals engaged in ≤2 hr regular natural physical activity^.^week^-1^. Hundred and seventy-nine late middle-aged (55–64 yrs = 67, 31 females and 36 males), young-old (65–74 yrs = 61, 24 females and 37 males), and old adults (75–84 yrs = 51, 21 females and 30 males) volunteers took part to this study. Fifty-six were senior athletes (55–64 = 27; 65–74 = 16; 75–84 = 13), 58 physically active (55–64 = 18; 65–74 = 22; 75–84 = 18), and 65 sedentary (55–64 = 22; 65–74 = 23; 75–84 = 20).

### Anthropometric evaluation

With participants wearing light underwear and no shoes, standing height to the nearest 0.1 cm and body mass to the nearest 0.1 kg, were measured using a portable stadiometer (Seca 220, GmbH & Co., Hamburg, Germany) and a balance scale (Seca 761, GmbH & Co., Hamburg, Germany), respectively. Body mass index (BMI, kg^.^m^-2^) was calculated to classify the participants according to the World Health Organization BMI cut-off points [2] into under-weight (<18.5 kg^.^m^-2^), normal-weight (range: 18.5–24.9 kg^.^m^-2^), overweight (range: 25.0–29.9 kg^.^m^-2^) and obese (≥30 kg^.^m^-2^) categories, respectively.

### Questionnaires

Assessments took place individually under the supervision of an investigator, who specified that there were no right or wrong responses. Prior to the evaluation, each individual answered the AAHPERD (American Alliance for Health, Physical Education, Recreation, and Dance) exercise/medical history questionnaire [40] ascertaining their activity level, educational background, dietary habits, tobacco smoking, and alcohol consumption, medication use and history of physical activity. Then, participants individually completed four on-line questionnaires to assess symptoms of body image dissatisfaction, exercise dependence, abnormal eating attitudes, and functional physical and mental health. The instruments showed Cronbach alpha coefficients ranging from 0.78 to 0.88.

#### Body Image

To assess the individual’s body dissatisfaction in relation to body size, the Body Image Dimensional Assessment (BIDA) instrument was used. The BIDA assesses the subjective and emotional dimensions of body image by means of a neutral (i.e., not sex and not ethnic-related) silhouette-based scale [41]. Participants had to indicate their perceived and ideal body shape, the most appropriate body shape for their peers and the most appreciated body shape by the opposite sex. They were not limited to selecting numerical values corresponding to images appearing on the scale, but they could indicate intermediate values using a scale ranging from 1.8 to 5.2 for which there are no representative images. Thus, Body Dissatisfaction (BD), Sexual Body Dissatisfaction (SxBD), Comparative Body Dissatisfaction (CBD), and Body Dissatisfaction Index (BDI) in relation to body dimension were calculated, with BDIabx (Absolute Body Dissatisfaction Index) >30% being considered at risk of body image disorders [41].

#### Exercise Dependence

Exercise dependence was evaluated by means of the 21-item Exercise Dependence Scale (EDS-21), which is based on a 6-point Likert scale anchored at the lowest extreme (i.e., 1) to “never” and at the highest extreme (i.e., 6) to “always” [42]. It evaluates seven dimensions of exercise addiction: 1) need for increased amounts of exercise to achieve the desired effect (Tolerance); 2) withdrawal symptoms for exercise (i.e., anxiety, fatigue) unless the exercise volume is maintained (Withdrawal); 3) higher performed exercise volume than intended (Intention Effects); 4) unsuccessful efforts to reduce or control exercise volume or intensity (Lack of Control); 5) which refers to the high amount of time spent in active living as recreational physical activity (Time); 6) restriction of social, occupational, or recreational activities in favour of engagement in exercise (Reductions in Other Activities); 7) exercise maintenance in presence of persistent/recurrent psychological or physical (i.e., injury) problems (Continuance). A multidimensional maladaptive pattern of exercise is manifested by scores >14 pt for at least 3 of the above 7 dimensions. In this study the internal consistency score for the 7 dimensions was 0.91. Furthermore, the total EDS score was computed by summing the 21-items.

#### Eating Attitudes

To identify eating attitudes and concerns about body weight, the 26-item Eating Attitude Test (EAT-26) [43] was used. Based on a 4-point agreement scale (from 4 = strongly agree to 1 = strongly disagree), three factors are identified (i.e., Dieting, Bulimia and Food Preoccupation, and Oral Control). Participants with an EAT-26 total score >20 pt are considered at risk of clinical disorders [43].

#### Health Survey

To assess functional health and well-being from the participant’s point of view the Short Form Health Survey Version 2^®^ (SF-12v2) was used [44]. Validated for use in the USA and many European countries [45,46], the instrument consists of 12 questions covering eight health domains: 1) physical functioning, 2) role limitations due to physical problems, 3) bodily pain, 4) general health perception, 5) energy and vitality, 6) social functioning, 7) role limitations due to emotional problems and 8) mental health. The eight scores are aggregated into two summary measures ranging from 0 (i.e., lowest level of health) to 100 (i.e., highest level of health): the Physical Component Summary (PCS) and the Mental Component Summary (MCS).

### Statistical analysis

Data were analysed using the Statistical Package for the Social Science, version 23.0 (SPSS Inc., Chicago Illinois).

First, a 3 x 3 x 2 multivariate analysis of variance (MANOVA) was applied to ascertain activity level (athletes, physically active, sedentary) and age class (55–64, 65–74, 75–84 yrs) differences, controlling for gender, in diet-related, and physical activity-related characteristics and attitudes and in health-related quality of life perception. The studied variables regarded anthropometry (BMI); body image (BD, SxBD, CBD, BDI); Exercise Dependence (Tolerance, Withdrawal, Intention Effects, Lack of Control, Time, Reductions in Other Activities, Continuance); Eating Attitudes (Dieting, Bulimia and Food Preoccupation, Oral Control); Health Survey (PCS, MCS). When significant differences emerged that needed multiple post-hoc comparisons, planned pairwise t-tests with Bonferroni corrections were used. Cohen’s d effect sizes (ES) were calculated for all significant findings [47].

Second, a mediation analysis was applied to assess whether the expected relationship between activity level and physical or mental health-related quality of life perception was explained by mechanisms that involved diet-related and physical activity-related personal characteristics and behaviours. Based on the hypothesized mediational chain that moves from physical activity and eating habits to their direct outcomes on weight status which, in turn, may impact subjective feelings of body (dis)satisfaction and related (un)healthy exercise behaviours, we run two serial multiple models (one for PCS and one for MCS) using the SPPS macro PROCESS. Thus, we evaluated the effect of: (1) the independent variable (X: activity level) on the dependent variable (Y: PCS or MCS); (2) the independent variable (X) on each mediator (M: total EAT score, BMI, BDI, or total EDS score); (3) the independent variable (X) and the potential mediators (M) on the dependent variable (Y). Then, bootstrapping was applied to empirically estimate the sampling distribution of the indirect effect and generate a bootstrap confidence interval (95% CI) based on 10,000 bootstrap samples for bias corrected bootstrap CIs. This CI was used as a form of hypothesis test to estimate if the size of the indirect effect of each individual mediator was different from zero [48].

## Results

### Activity level, age and gender influence on the study variables

Table 1 reports the anthropometric characteristics, regular use of medications, number of diseases, and education background of the participants. As regards anthropometric measures, main effects on BMI were found for activity level (*F*_(2,176)_ = 13.503, *p*<0.001) and gender (*F*_(1,177)_ = 13.442, *p*<0.001, ES = 0.4). Regarding activity level, post hoc analyses revealed that athletes differed from physically active and sedentary counterparts (*p* = 0.001, ES = 0.7 and *p*<0.001, ES = 0.9, respectively). Overall, BMI resulted lowest for athletes, intermediate for physically active individuals, and highest for sedentary individuals (24.0±2.7, 27.3±3.8 and 28.1±4.1 kg^.^m^2^, respectively).

**Table 1:** Anthropometric characteristics, weight category, number of medications and diseases, and educational background of participants.

		Athlete			Physically Active			Sedentary		
	Gender	55–64	65–74	75–84	55–64	65–74	75–84	55–64	65–74	75–84
**Anthropometry**										
Body mass (kg)	F	64.3±8	55.4±1.7	63.2±4.2	70.7±11.2	65.6±9	66.9±8.9	66.3±12.1	68±11.1	59.1±6.3
	M	80.6±11.3	80.5±9.2	71±7	90.1±12	77.6±10.4	77.5±8.2	91±13.5	86.2±8.9	74.6±9.9
Height (m)	F	1.65±0.05	1.65±0.04	1.60±0.06	1.64±0.06	1.55±0.05	1.58±0.06	1.59±0.04	1.56±0.04	1.53±0.08
	M	1.76±0.07	1.74±0.06	1.71±0.09	1.78±0.04	1.68±0.06	1.69±0.05	1.72±0.09	1.70±0.07	1.67±0.07
BMI (kg∙m^-2^)	F	23.6±2.9	20.4±0.4	23.3±2.9	26.5±4.8	27.3±3.5	26.9±4.4	26.3±4.3	27.9±3.6	25.3±3.2
	M	26±2.8	26.5±2	24.4±1.4	28.3±3.5	27.6±3.2	27.2±3.9	31±5.1	29.8±2.7	26.8±2.1
**Weight category**										
Normal weight (%)	F	26	8	5	13	8	8	10	8	15
	M	25	14	21	7	14	7	4	0	7
Overweight (%)	F	7	0	4	11	18	21	18	14	7
	M	12	15	8	8	10	10	8	15	15
Grade I Obesity (%)	F	0	0	0	0	38	0	13	38	13
	M	10	5	0	15	15	0	25	25	5
Grade II Obesity (%)	F	0	0	0	100	0	0	0	0	0
	M	0	0	0	0	0	50	50	0	0
Grade III Obesity (%)	F	0	0	0	0	0	0	0	0	0
	M	0	0	0	0	0	0	100	0	0
**Health**										
Medications (n)	F	1.3±1	0±0	1.3±1.5	3.4±2.5	5±3	4.3±4.6	2.4±1.7	5.4±3.3	4.6±4.1
	M	1.2±1.3	1.6±1.6	2.3±1.3	1.3±1.5	3.7±1.6	4.6±2.8	3.2±2.6	3.2±1.8	4.3±3
Diseases (n)	F	0.8±1.1	0±0	0.7±0.6	1.7±1.9	2.5±2.3	4.3±2	2.1±2.6	3.4±2.5	5.4±2.7
	M	0.5±1.1	2.2±2.2	2.1±2	1.2±1.8	3.1±2.2	4.3±2	2.3±2.6	2.8±2.6	2.5±3.3
**Educational background**										
College (%)	F	40	0	5	15	0	15	20	5	0
	M	13	19	13	9	9	6	9	6	16
High school (%)	F	11	8	3	14	22	14	14	8	8
	M	20	10	8	13	15	3	10	15	8
<High school (%)	F	0	0	5	5	16	5	5	32	32
	M	10	10	10	3	10	16	16	16	10

Regular use of medications and number of diseases showed a main effect for activity level (medications: F_(2,176)_ = 14.448, *p*<0.001; diseases: F_(2,176)_ = 10.645, *p*<0.001) and for age class (medications: F_(2,176)_ = 4.909, *p* = 0.009; diseases: F_(2,176)_ = 8.568, *p*<0.001). Post hoc analyses revealed lower medication and disease reported by athletes than physically active or sedentary counterparts (medications: *p*<0.001, ES = 1.0 and *p*<0.001, ES = 1.1; diseases: *p* = 0.001, ES = 0.8, and *p*<0.001; ES = 0.7, respectively), and by 55–64 year-olds than their 65–74 and 75–84 year-old counterparts (medications: *p* = 0.005, ES = 0.6 and *p* = 0.001, ES = 0.7; diseases: *p* = 0.006, ES = 0.6 and *p*<0.001; ES = 0.9, respectively).

Regarding body dissatisfaction (Table 2), a main effect was found for activity level with respect to BD (*F*_(2,176)_ = 5.654, *p* = 0.004), SxBD (*F*_(2,176)_ = 6.391, *p* = 0.002), and CBD (*F*_(2,176)_ = 13.002, *p*<0.001), and for age class with respect to CBD: (*F*_(2,176)_ = 5.321, *p* = 0.006). Post hoc analyses showed that athletes differed from both physically active and sedentary counterparts in BD (*p* = 0.018, ES = 0.5 and *p* = 0.001, ES = 0.7) and CBD (*p* = 0.028, ES = 0.6 and *p*<0.001, ES = 0.9, respectively), but only from sedentary individuals in SxBD (*p*<0.001, ES = 0.7). As regards the age class effect on CBD, there was a significant difference (*p* = 0.029, ES = 0.5) only between 55–64 and 75–84 year-old individuals.

**Table 2:** BIDA indexes in relation to activity level, age class, and gender (mean±SD).

	BD	SxBD	CBD	BDIabx
**Activity level**				
Athlete	8.9±14.4*	5.1±21.5^#^	-20.5±18.5*	17.5±8.6
Physically Active	16.3±13.1	13.6±18.4	-11.1±16.7	16.8±8.2
Sedentary	18.6±14.8	19.9±19.5	-3.2±22.6	19.8±10.5
**Age class**				
55–64	16.6±15.3	16±20.6	-7±22.2^$^	19.2±10.7
65–74	15.8±15.1	15.4±20.4	-11.5±22.3	19.2±8.3
75–84	11.4±12.8	6.9±19.9	-16.3±15.3	15.3±7.8
**Gender**				
Female	16.5±14.6	13.6±19.1	-9.5±20.5	17.6±9.8
Male	13.6±14.7	12.9±21.7	-12.4±20.9	18.5±8.9

* = P<0.05 with respect to physically active and sedentary

# = P<0.05 with respect to sedentary

$ = P<0.05 with respect to 75–84 age class

A risk of body image disorders (BDI >30%) [41] was observed in 5 athletes (1 female and 4 males), 5 physically active (3 females, 2 males) and 9 sedentary participants (4 females and 5 males). Considering their objective weight status, these individuals at risk of body image disorder were distributed as follows: 2 normal weight males in the athletes category and 2 normal weight females in the sedentary category; 2 overweight (1 female, 1 male) in the physically active category; 12 obese (4 females, 8 males) individuals in all three activity categories (1 obese I grade athlete, 3 obese I grade and 1 obese II grade physically actives, 6 obese I grade and 1 obese II grade sedentary individuals).

Regarding the EDS-21 questionnaire (Table 3), a main effect for activity level was found for Tolerance (*F*_(2,176)_ = 67.504, *p*<0.001), Withdrawal (*F*_(2,176)_ = 18.487, *p*<0.001), Intention Effects (*F*_(2,176)_ = 30.896, *p*<0.001), Lack of Control (*F*_(2,176)_ = 28.982, *p*<0.001), Time (*F*_(2,176)_ = 57.995, *p*<0.001), Reduction in Other Activities (*F*_(2,176)_ = 11.936, *p*<0.001), and Continuance (*F*_(2,176)_ = 31.009, *p*<0.001), with athletes showing highest values, physically actives intermediate values and sedentary individuals lowest values. For all the EDS-21 dimensions, post hoc analyses revealed significant differences between athletes and sedentary counterparts (*p*<0.001, ES = 0.8–2.2), with overall higher values for athletes. Athletes also scored significantly higher than physically active individuals and the latter higher than sedentary counterparts in Tolerance, Withdrawal, Intention Effects, and Time (*p*≤0.01, ES = 0.5–1.2). Moreover, athletes and physically active individuals differed in Reduction in Other Activities (*p* = 0.004, ES = 0.5), whereas physically active and sedentary individuals differed in Lack of Control and Continuance (*p* = 0.002, ES = 1.1, and *p*<0.001, ES = 1.2, respectively). Finally, risk of exercise addiction (scores >14 pt) [42] was found only in 6 athletes (2 female swimmers, in the 55–64 and 75–84 age classes, respectively; 3 male swimmers in the 55–64 and 65–74 age classes, respectively; and 1 male runner in the 55–64 age class).

**Table 3:** EDS-21 dimensions in relation to activity level and gender (mean±SD).

	Tolerance	Withdrawal	Intention Effects	Lack of Control	Time	Reduction in Other Activities	Continuance
**Activity level**							
Athlete	11.6±4.2*	8.1±4.5*	7.5±3.6*	8.4±4.5^#^	10.4±4*	6.4±3.6*	8.6±4.7^#^
Physically Active	7±3.5^§^	6.2±3.7^§^	5.5±2.7^§^	6.9±3.9^§^	7.8±3.2^§^	4.8±2.2	7.4±3.6^§^
Sedentary	4.1±2.4	4.3±2.2	3.7±1.6	3.6±1.5	4±1.8	4.1±1.7	3.8±1.9
**Gender**							
Female	6.8±4.5	6.5±4.4^†^	4.9±2.9	6.1±4	6.7±3.8	4.8±2.8	6.4±4.1
Male	7.8±4.6	5.8±3.4	5.8±3.3	6.2±4	7.7±4.2	5.2±2.7	6.5±4.1

* = P≤0.01 with respect to physically active and sedentary

# = P≤0.01 with respect to sedentary

§ = P≤0.01 with respect to sedentary

† = P<0.05 with respect to male

Regarding the EAT-26 questionnaire (Table 4), a main effect for activity level was found only with respect to Oral Control (*F*_(2,118)_ = 3.358, *p* = 0.023). Post hoc analysis revealed higher values for athletes than sedentary counterparts (*p* = 0.002, ES = 0.3). Furthermore, an activity level x age class interaction was found for Bulimia and Food Preoccupation (*F*_(4,161)_ = 2.761, *p* = 0.03). Post hoc analysis revealed a difference for 65–74 year-old individuals only (*F*_(2,58)_ = 6.016, *p* = 0.004), with athletes showing lower values (0.69±0.87 pt) than sedentary individuals (2.17±1.23 pt; *p* = 0.004, ES = 1.4).

**Table 4:** EAT-26 factors in relation to activity level and gender (mean±SD).

	Dieting	Bulimia and Food Preoccupation	Oral Control
**Activity level**			
Athlete	2.5±3.6	1.6±1.8	1.5±1.8^#^
Physically Active	3.8±4.5	1.5±1.4	1.0±1.7
Sedentary	2.5±3.2	1.7±1.3	0.6±1.2
**Gender**			
Female	3.1±4.2	1.7±1.6	0.7±1.3
Male	2.8±3.5	1.5±1.4	1.3±1.8

# = P<0.05 with respect to sedentary

### Analysis of determinants of mental and physical health perception

In the MANOVA performed on the variables derived from the SF-12 questionnaire (Table 5), a main effect for activity level emerged for both physical and mental components of health-related quality of life perception (PCS: *F*_(2,176)_ = 6.604, *p* = 0.002; MCS: *F*_(2,176)_ = 3.846, *p* = 0.023). Athletes showed highest MCS and PCS scores, physically actives the intermediate scores, and sedentary individuals lowest scores. Post hoc analysis revealed significant differences only between athletes and sedentary counterparts (PCS: *p* = 0.001, ES = 0.7; MCS: *p* = 0.015, ES = 0.5).

**Table 5:** SF-12 components in relation to activity level, age class, and gender mean±SD).

	PCS	MCS
**Activity level**		
Athlete	54.6±4.4^#^	53.6±6.8^#^
Physically Active	51.7±7.3	51.3±9.3
Sedentary	49.7±8.3	49.3±9.9
**Age class**		
55–64	52.9±6.7	48.8±10.5^$^
65–74	50.1±7.6	51.9±7.6
75–84	52.7±7	53.8±7.5
Gender		
Female	51.2±7.4	49.4±10.2
Male	52.4±7.1	52.7±7.7

# = P<0.05 with respect to sedentary

$ = P<0.01 with respect to 75–84 age class

A main effect for age emerged for MCS only (*F*_(2,176)_ = 6.086, *p* = 0.003). Post hoc analysis revealed that 55–64 year-old individuals scored significantly lower than their 75–84 year-old counterparts (*p* = 0.004, ES = 0.6). Furthermore for MCS, a gender x age class interaction was found (*F*_(2,176)_ = 3.520, *p* = 0.032). Post hoc analysis revealed an age-related difference for female individuals only (*p* = 0.005), with 55–64 year-old females scoring lower (45.1±11.2 pt) than their 75–84 old counterparts (53.8±6.5 pt; *p* = 0.006, ES = 1.0).

### Analysis of mechanisms underlying the relation of activity level to health perception

**Fig 1:**
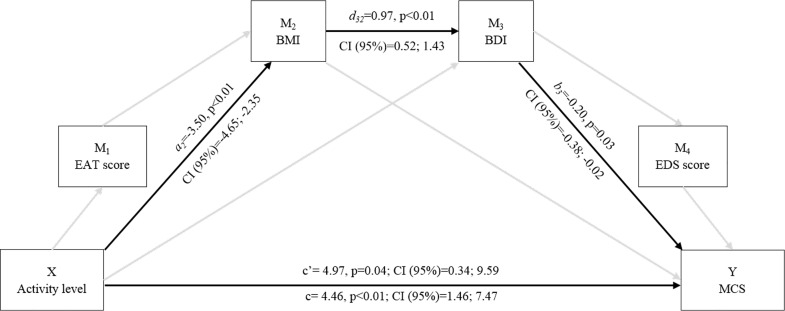
Conceptual and statistical model of the activity level and mediators effect’s on MCS. c’ = direct effect; c = total effect; a_2·_d_32·_b_3 =_ indirect effect through BMI and BDI.

Since the MANOVA performed on the variables of perceived health-related quality of life in the physical and mental domain revealed significant differences only between athletes and sedentary individuals, mediation analysis was performed on data of athletes and sedentary individuals only, to test whether diet- and/or physical activity-related characteristics and attitudes were mechanism underlying the activity-perceived health relationship. The mediation analysis showed the existence of a significant mediation path by BMI and BDI variables only in the case of MCS (Fig 1), but not in the case of PCS. This is indicated by the bootstrapping output: only in the case of the serial indirect effect path including BMI and BDI as mediators, the 95% CI of bootstrap estimates of the indirect effect did not include the zero value (0.69, Bootstrap CI = 0.12; 1.91). Instead, the bootstrap CI for all the others indirect effects did not allow to exclude the null hypothesis and are not reported in the figure. Thus, eating attitudes and exercise dependence variables did not significantly contribute to the serial mediation path linking activity habits to body weight and image variables and, lastly, to perceived mental health scoring.

## Discussion

The present study aimed at furthering our understanding of (i) the relationship between type of physical activity habits and perceived health-related quality of life in aging and (ii) the potential mechanisms underlying this relationship in the mental and physical domains. The search for mediating mechanisms has been previously acknowledged as relevant for both policymakers and implementation professionals [19]. To our knowledge, this is the first study exploring the complex mechanisms underlying the associations between an active lifestyle and perceived health in the physical and mental domains in aging by means of multifaceted measures focusing on interrelated behavioural nutrition and physical exercise attitudes/disorders, as objectification, body image concerns, eating, and exercise attitudes. Findings highlight the crucial role of physical activity habits in determining both physical and mental health-related quality of life perceptions with advancing age. Moreover, they suggest that there are different pathways through which a physically active lifestyle translates into perceived health and quality of life, with body mass and (dis)satisfaction mediating the relation of a prolonged history of sport activity to perceived mental health, but not to perceived physical health.

### Health-related quality of life, body mass and image, eating and exercise attitudes in aging: commonalities and differences between athletes, physically actives, or inactives

Consistent with the literature showing an overwhelming positive association of an active lifestyle with the reduction of preventable chronic diseases and increased self-perceived quality of life in older individuals [20], our results showed that being physically active is associated to reduced occurrence of pathologies and number of medication and higher perceived physical and mental health. This association was independent of the opposite, negative effect of age. While descriptive statistics suggested general trends of best health outcomes of extensive sport practice, intermediate outcomes of habitual physical activity and worst outcomes of sedentary habits, significant differences were observed between athletes and sedentary counterparts only. Conversely, individuals involved in structured physical activity and their sedentary counterparts did not significantly differ in diseases, medication, and perceived health, in line with the lack of evidence for a robust association between general health and physical activity [23]. Indeed, it has been claimed that physical activities may vary in terms of aims, quality and quantity, thus determining different outcomes and health effects in relation to the individual’s capabilities, needs and preferences [49]. Our findings substantiate the knowledge that commitment to competitive sport in later life contributes to successful aging [50], suggesting that sport involvement that requires staying active above the recommended levels provides additional benefits beyond those derived from general engagement in physical activity.

Furthermore, only senior athletes who maintained a desirable body mass reported better perceived body image and health than sedentary or physically active counterparts not committed to sport. Aging determines several bodily changes, including increases in body weight, body fat and fat distribution, which may affect physical and mental health of older individuals [7]. In line with the literature [51], engagement in competitive sport resulted positive for maintaining normal weight with advancing years, although a gender difference in frequency of lean athletes emerged in favour of females according to gender stereotypes of appearance and thinness [52]. The higher percentage of overweight men among athletes can be also explained referring to the claim that the ‘optimal’ BMI category for older fit individuals could be the overweight one, probably reflecting the maintenance of muscle mass due to chronic exercise. [53,54].

There is a general consensus that older individuals are dissatisfied with their bodies. However, when controlling for exercise frequency, duration, intensity, and typology [34], exercise training seems associated with improved body image. In the present study, senior athletes reported a better body image than physically active and sedentary co-aged individuals, probably because athletes tend to resemble the aesthetic ideal of a healthy and appealing physique. Conversely, the lack of differences between sedentary and physically active subgroups might be due to the fact that exercise programs for older adults often include moderate intensity exercises, which might not have an effect on perceived body image [34]. Overweight and obese individuals tended to be at risk of body image disorders, confirming that an excessively high BMI is at odds with the societal demands to conform to a lean body-type.

Problematic eating [55] and exercise attitudes [56] have been reported in youth athletes engaging in competitive sports that require the adoption of intense training programmes and specific diets to control body weight and to ensure athletic performances. Conversely, there is a paucity of information on potential negative experiences and outcomes of participation in sport and exercise programmes in older individuals [57].

In our study with late middle-aged and older adults, independently of their activity level, no participants resulted at risk of clinical eating disorders [43]. Although senior athletes showed significantly higher scores of Oral Control than sedentary counterparts, their values remained well insufficient to mirror eating disorders. Furthermore, contrarily to younger samples [55], no significant gender differences were evident in our sample of elderly except for Oral Control where females scored lower than males. It can be suggested that physical activity has taken the place of dieting in controlling BMI among older women.

Senior athletes scored higher in all dimensions of the Exercise Dependence Scale than their physically active and sedentary counterparts, with 11% of senior athletes resulting at risk of exercise addiction. This novel finding highlights the other side of the coin of the health-related benefits of competitive sport participation, indicating that even at older age, sport commitment at national and international level requiring large amounts of training to pursue outstanding sport performances represents a risk for maladaptive exercise behaviours similar to what observed with younger athletes [56]. However, to properly interpret the present finding of a non-negligible percentage of senior athletes at risk of exercise dependency as one’s exercise obsession rather than healthy passion for sport, there is a need of an in-depth scrutiny to ascertain the presence of loss of control over the exercise behaviour with consequent negative physical and psychosocial outcomes. In fact, a nomothetic research approach could not distinguish the several combinations of subjective psychological factors and situational variables interactively determining an exercise addiction [56].

### A three-path mediation link between activity habits, body mass and image, and mental health

The novelty of the study is that beyond the general finding that senior athletes perceive better physical and mental health than sedentary co-aged individuals, there may be more direct or indirect paths linking a prolonged history of sport participation to the perception of a good health depending on the considered health domains. For physical health perception, there seems to be a more direct link to chronic sports participation. This is not surprising, since regular performance-oriented sports training considerably contributes to physical activity at health enhancing levels [58], thus strongly impacting those physical and motor fitness outcomes that are essential for physical functioning. Indeed, none of the four diet-related and exercise-related factors tested as potential mediators statistically weakened the direct relationship between chronic sports participation and perceived physical health.

A different picture emerged for mental health which is more complex in nature. Fig 1 shows how the relation to habitual sports participation is mediated by body mass and body image (dis)satisfaction. Whilst taken alone, body mass and body image did not show a significant effect on perceived mental health, they jointly affected it in a serial fashion. Having a long and still actual history of sport commitment, as compared to having a sedentary lifestyle, seems to contribute maintaining an objectively healthier body mass and, as a consequence, a subjectively more satisfactory body image, which in turn positively influences the perception of mental health. These results are in line with the increasing engagement of old individuals in sport who want to contrast the stereotyped image of frail, dependent, and lonely older individuals [57].

It is interesting that not pathological behavioural nutrition and exercise attitudes, but body mass and its effect on body image resulted a joining link between chronic sport participation and perceived mental health. Usually, studies have independently linked physical (in)activity and weight status to mental health outcomes, particularly cognitive function [22,59]. Recently, there is a renewed attention for the interrelations between physical activity, overweight, cognitive functioning, and mental health-related quality of life perception [59–61]. Weight status is a candidate mediator of physical activity effects on cognitive functioning [62]. To explain this mediation path, neuro-metabolic explanations have been proposed referring to the linkage between exercise-related physical fitness, overweight and their opposite effects on brain health and cognitive performance [60]. Our results suggest the existence of a more psychological mediation mechanism, which explains the association between habitual sport participation and mental health perception referring to the linkage between healthy weight status and positive cognitive-emotional dimensions of the body image construct [29].

Limitations of this study include the cross-sectional nature of the data and the classification of the activity level which was based on a subjective rather than an objective evaluation. Future studies should be conducted with longitudinal and interventional data and using objective measurements (i.e., pedometer or accelerometer) of the activity level.

## Conclusions

This study revealed the crucial role of an active lifestyle and habitual sport participation in determining physical and mental health perception at advanced age. The employed broad focus on body mass and image satisfaction, eating, and exercise attitudes allowed identifying a mediational chain that furthers our understanding of the physical activity-mental health relationship. Measuring individual mediator candidates seems not sufficient to study the such complex relationship. In conclusion, our results support the view that senior athletes provide insights in the negotiation of the aging process beyond the general recommendations on the amount of physical activity needed for health promotion and maintenance [57]. This kind of evidence, helps understand how and why lifelong sport participation contributes to a relevant public health goal as maintaining a good health and quality of life perception at advanced age [63] This may be useful to substantiate physical activity promotion policies that join organizational goals and obligations of the sports and health sectors [64].

## Supporting Information

S1 Dataset Anthropometric and questionnaires data. (XLSX)

S1 Appendix English version of the questionnaires. (PDF)

S2 Appendix Italian version of the questionnaires. (PDF)
