# Supplementary material for: Physical Activity and Health Perception in Aging: Do Body Mass and Satisfaction Matter? A Three-Path Mediated Link
Source: PLoS One. 2016 Sep 9;11(9):e0160805. doi: 10.1371/journal.pone.0160805 (PMC5017576; doi:10.1371/journal.pone.0160805)
Supplement: S1 Appendix — (PDF) [file pone.0160805.s002.pdf]

Università degli Studi di Roma "Foro Italico"

* Required

QUESTIONNAIRES

The following questionnaires evaluate your health status, physical activity level, perception of body image, eating behavior, exercise attitude. There are no right or wrong answers. Fill out all the questionnaires carefully and honestly. All answers are confidential.

1. **Identification code** *

____

2. **Birthday:** *
(dd/mm/yyyy)

____

3. **Are you in retirement?** *

- [ ] Yes
- [ ] No

4. **If you answered NO, indicate your job:**

____

5. **Indicate your educational level:** *

- [ ] Primary school
- [ ] Middle school
- [ ] High school
- [ ] Bachelor
- [ ] Master
- [ ] Other ________________________________

6. **Body mass:** *

____

7. **Height:** *

____

Physical health status

8. **List the reasons why you are hospitalized in the past 5 years:**

____
____
____

9. **List the diseases recently had:**

____
____
____

10. **Indicate the health problems:**

- [ ] Anemia
- [ ] Arthritis, bursitis
- [ ] Asthma
- [ ] High pressure
- [ ] Low pressure
- [ ] Chest pains
- [ ] Intestinal problems
- [ ] Bladder problems
- [ ] Discomfort during exercise
- [ ] Diabetes
- [ ] Difficulty hearing
- [ ] Difficulty seeing
- [ ] Dizziness
- [ ] Heart conditions

- [ ] Hernia
- [ ] Indigestion
- [ ] Joint pain
- [ ] Leg pain during walking
- [ ] Respiratory problems, shortness of breath
- [ ] Osteoporosis
- [ ] Lower back pain
- [ ] High cholesterol
- [ ] Gastric problems
- [ ] Other conditions

11. **Smoking status:** *

- [ ] I have smoked in past
- [ ] I do not smoke
- [ ] I smoke now

12. If you answered I smoke now, indicate how many cigarettes per day

____

13. **Alcohol consumption:** *

- [ ] Never
- [ ] Occasionally

14. If you answered Occasionally, indicate how many glasses per day

____

15. **List health problems due to alcohol consumption:**

____
____
____

16. **Medical prescriptions:** *

- [ ] No
- [ ] Yes

17. **If you answered Yes, indicate the medical prescriptions (name and dose):**

____
____
____

18. **Do you use supplements? List them:** *

____
____
____

Physical activity level

19. **How do you evaluate your physical activity level during the last year?** *

- [ ] LOW: sitting, bending, driving, talking, no pre-planned exercise
- [ ] MEDIUM: standing, walking, bending, moving
- [ ] MODERATE: standing, walking, bending, moving, exercise once per week
- [ ] ACTIVE: light exercise, stairs climbing, exercise 2-3 times per week
- [ ] VERY ACTIVE: moderate exercise, regular exercise 4 times per week or more

20. **Physical activity or sport practiced:** *

- [ ] Nothing
- [ ] Gym
- [ ] Running

- [ ] Swimming
- [ ] Cycling
- [ ] Basket
- [ ] Soccer
- [ ] Golf
- [ ] Tennis
- [ ] Track and field
- [ ] Other: ___________________________

21. **How many times per week do you exercise?** *

- [ ] 0
- [ ] 1
- [ ] 2
- [ ] 3
- [ ] 4
- [ ] 5
- [ ] 6
- [ ] 7

22. **How many hours per week do you exercise?** *

- [ ] 0
- [ ] 1 – 2 hours
- [ ] 2 – 3 hours
- [ ] 3 – 6 hours

- [ ] More than 6 hours

23. **How long does each session take?** *

- [ ] No exercise
- [ ] Less than 1 hour
- [ ] 1 – 2 hours
- [ ] 2 – 3 hours
- [ ] More than 3 hours
- [ ] Other: ___________________________

24. **Do you have a trainer?** *

- [ ] Yes
- [ ] No

25. **Which kind of activity do you practice?** *

- [ ] No activity
- [ ] Recreational/amateur activity
- [ ] Competitive activity

26. **If you answered Competitive activity, indicate how many competitions per month**:

____

BODY IMAGE DIMENSIONAL ASSESSMENT (BIDA)

Referring to the figure below, answer to the following questions using a scale from 1.8 to 5.2 for the perception of body image dimension. Intermediate values are allowed (e.g., 3.6).

Body Image Dimensional Assessment (BIDA)

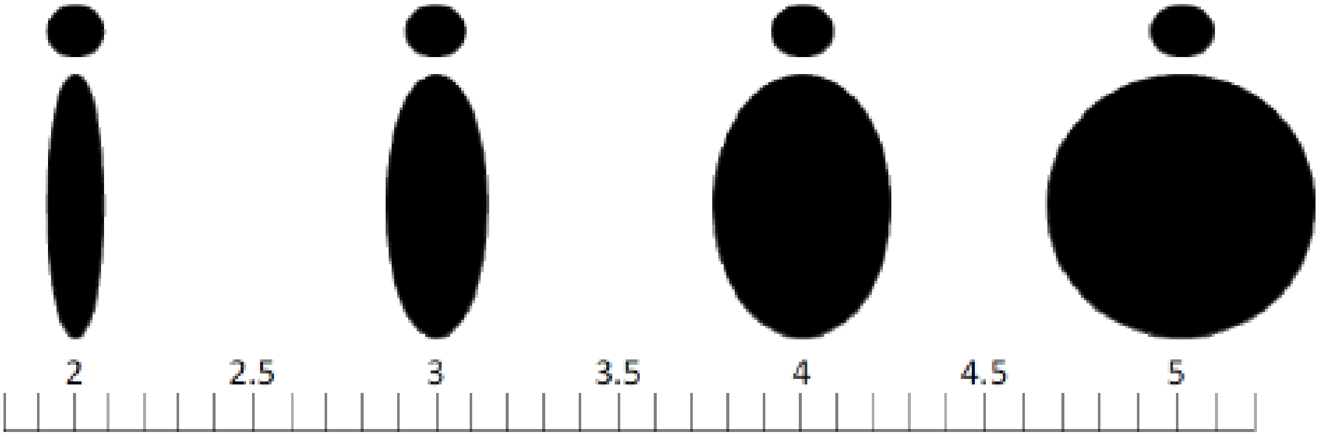

1. What do you think you look like currently?
   *Answer*________________

2. Which is your ideal figure?
   *Answer*________________

3. Which is the most attractive figure for the opposite sex?
   *Answer*________________

4. How do most people with your own sex and age look like?
   Answer ________________

Could you indicate your weight and your height?

Weight ____________ Height_____________

Age _______________ Gender ______________

(For the interviewer: Weight ______________ Height_______________)

EATING ATTITUDES TEST (EAT26)

Eating Attitudes Test (EAT-26)©

Instructions: This is a screening measure to help you determine whether you might have an eating disorder that needs professional attention. This screening measure is not designed to make a diagnosis of an eating disorder or take the place of a professional consultation. Please fill out the below form as accurately, honestly and completely as possible. There are no right or wrong answers. All of your responses are confidential.

Part A: Complete the following questions:

1) Birth Date	Month:		Day:		Year:		2) Gender:	Male	Female
3) Height	Feet :		Inches:					☐	☐
4) Current Weight (lbs.):			5) Highest Weight (excluding pregnancy):						
6) Lowest Adult Weight:			7: Ideal Weight:						

	**Part B: Check a response for each of the following statements:**	Always	Usually	Often	Some times	Rarely	Never
1.	Am terrified about being overweight.	☐	☐	☐	☐	☐	☐
2.	Avoid eating when I am hungry.	☐	☐	☐	☐	☐	☐
3.	Find myself preoccupied with food.	☐	☐	☐	☐	☐	☐
4.	Have gone on eating binges where I feel that I may not be able to stop.	☐	☐	☐	☐	☐	☐
5.	Cut my food into small pieces.	☐	☐	☐	☐	☐	☐
6.	Aware of the calorie content of foods that I eat.	☐	☐	☐	☐	☐	☐
7.	Particularly avoid food with a high carbohydrate content (i.e. bread, rice, potatoes, etc.)	☐	☐	☐	☐	☐	☐
8.	Feel that others would prefer if I ate more.	☐	☐	☐	☐	☐	☐
9.	Vomit after I have eaten.	☐	☐	☐	☐	☐	☐
10.	Feel extremely guilty after eating.	☐	☐	☐	☐	☐	☐
11.	Am preoccupied with a desire to be thinner.	☐	☐	☐	☐	☐	☐
12.	Think about burning up calories when I exercise.	☐	☐	☐	☐	☐	☐
13.	Other people think that I am too thin.	☐	☐	☐	☐	☐	☐
14.	Am preoccupied with the thought of having fat on my body.	☐	☐	☐	☐	☐	☐
15.	Take longer than others to eat my meals.	☐	☐	☐	☐	☐	☐
16.	Avoid foods with sugar in them.	☐	☐	☐	☐	☐	☐
17.	Eat diet foods.	☐	☐	☐	☐	☐	☐
18.	Feel that food controls my life.	☐	☐	☐	☐	☐	☐
19.	Display self-control around food.	☐	☐	☐	☐	☐	☐
20.	Feel that others pressure me to eat.	☐	☐	☐	☐	☐	☐
21.	Give too much time and thought to food.	☐	☐	☐	☐	☐	☐
22.	Feel uncomfortable after eating sweets.	☐	☐	☐	☐	☐	☐
23.	Engage in dieting behavior.	☐	☐	☐	☐	☐	☐
24.	Like my stomach to be empty.	☐	☐	☐	☐	☐	☐
25.	Have the impulse to vomit after meals.	☐	☐	☐	☐	☐	☐
26.	Enjoy trying new rich foods.	☐	☐	☐	☐	☐	☐

	**Part C: Behavioral Questions:<br>In the past 6 months have you:**	Never	Once a month or less	2-3 times a month	Once a week	2-6 times a week	Once a day or more
A	Gone on eating binges where you feel that you may not be able to stop? *	☐	☐	☐	☐	☐	☐
B	Ever made yourself sick (vomited) to control your weight or shape?	☐	☐	☐	☐	☐	☐
C	Ever used laxatives, diet pills or diuretics (water pills) to control your weight or shape?	☐	☐	☐	☐	☐	☐
D	Exercised more than 60 minutes a day to lose or to control your weight?	☐	☐	☐	☐	☐	☐
E	Lost 20 pounds or more in the past 6 months	Yes ☐		No ☐			

* Defined as eating much more than most people would under the same circumstances and feeling that eating is out of control

© Copyright EAT-26: (Garner et al. 1982, *Psychological Medicine*, 12, 871-878); adapted by D. Garner with permission.

EXERCISE DEPENDENCE SCALE21

EDS-21

Hausenblas & Symons Downs (2002)

Instructions. Using the scale provided below, please complete the following questions as honestly as possible. The questions refer to current exercise beliefs and behaviors that have occurred in the past 3 months. Please place your answer in the blank space provided after each statement.

	**1 Never**	**2**	**3**	**4**	**5**	**6 Always**
1. I exercise to avoid feeling irritable.	☐	☐	☐	☐	☐	☐
2. I exercise despite recurring physical problems.	☐	☐	☐	☐	☐	☐
3. I continually increase my exercise intensity to achieve the desired effects/benefits.	☐	☐	☐	☐	☐	☐
4. I am unable to reduce how long I exercise.	☐	☐	☐	☐	☐	☐
5. I would rather exercise than spend time with family/friends.	☐	☐	☐	☐	☐	☐
6. I spend a lot of time exercising.	☐	☐	☐	☐	☐	☐
7. I exercise longer than I intend.	☐	☐	☐	☐	☐	☐
8. I exercise to avoid feeling anxious.	☐	☐	☐	☐	☐	☐
9. I exercise when injured.	☐	☐	☐	☐	☐	☐
10. I continually increase my exercise frequency to achieve the desired effects/benefits.	☐	☐	☐	☐	☐	☐
11. I am unable to reduce how often I exercise.	☐	☐	☐	☐	☐	☐
12. I think about exercise when I should be concentrating on school/work.	☐	☐	☐	☐	☐	☐
13. I spend most of my free time exercising.	☐	☐	☐	☐	☐	☐
14. I exercise longer than I expect.	☐	☐	☐	☐	☐	☐
15. I exercise to avoid feeling tense.	☐	☐	☐	☐	☐	☐
16. I exercise despite persistent physical problems.	☐	☐	☐	☐	☐	☐
17. I continually increase my exercise duration to achieve the desired effects/benefits.	☐	☐	☐	☐	☐	☐
18. I am unable to reduce how intense I exercise.	☐	☐	☐	☐	☐	☐
19. I choose to exercise so that I can get out of spending time with family/friends.	☐	☐	☐	☐	☐	☐
20. A great deal of my time is spent exercising.____	☐	☐	☐	☐	☐	☐
21. I exercise longer than I plan.	☐	☐	☐	☐	☐	☐
