# Supplementary material for: Physical Activity and Health Perception in Aging: Do Body Mass and Satisfaction Matter? A Three-Path Mediated Link
Source: PLoS One. 2016 Sep 9;11(9):e0160805. doi: 10.1371/journal.pone.0160805 (PMC5017576; doi:10.1371/journal.pone.0160805)
Supplement: S2 Appendix — (PDF) [file pone.0160805.s003.pdf]

# Università degli Studi di Roma "Foro Italico"

* Required

## QUESTIONARI

I seguenti questionari sono indirizzati alla valutazione del suo stato di salute, del suo livello di attività fisica, della percezione della sua immagine corporea, del suo comportamento alimentare e della sua attitudine verso l'esercizio fisico. Non esistono risposte corrette o sbagliate. Compili completamente tutti i questionari in maniera accurata ed onesta. Tutte le risposte sono confidenziali.

1. **Codice identificativo** *

   ............................................................

2. **Data di nascita:** *

   (gg/mm/aaaa)

   ............................................................

3. **Sei in pensione?** *

   *Mark only one oval.*

   ( ) Si
   ( ) No

4. **Se hai risposto NO, indica la tua attività lavorativa:**

   ............................................................
   ............................................................
   ............................................................
   ............................................................
   ............................................................

5. **Indica il tuo livello di istruzione:** *

   *Mark only one oval.*

   ( ) Licenza elementare
   ( ) Licenza media
   ( ) Diploma
   ( ) Laurea
   ( ) Other: ............................................................

6. **Peso:** *

   ............................................................

7. **Statura:** *

   ............................................................

## Stato di salute fisica

8. **Elenca i motivi per cui ti sei ricoverato in ospedale negli ultimi 5 anni:**

.................................................................................

.................................................................................

.................................................................................

.................................................................................

.................................................................................

9. **Elenca le malattie avute recentemente:**

.................................................................................

.................................................................................

.................................................................................

.................................................................................

.................................................................................

10. **Indica i problemi di salute:**
*Check all that apply.*

- [ ] Anemia
- [ ] Artrite, borsiti
- [ ] Asma
- [ ] Pressione alta
- [ ] Pressione bassa
- [ ] Dolori al petto
- [ ] Problemi intestinali
- [ ] Problemi vescicali
- [ ] Disagio durante esercizio
- [ ] Diabete
- [ ] Difficoltà a sentire
- [ ] Difficoltà a vedere
- [ ] Vertigini
- [ ] Condizioni del cuore
- [ ] Ernia
- [ ] Indigestione
- [ ] Dolore alle articolazioni
- [ ] Dolore delle gambe durante camminata
- [ ] Problemi respiratori, respiro corto, fiatone
- [ ] Osteoporosi
- [ ] Dolori lombari
- [ ] Colesterolo alto
- [ ] Problemi gastrici
- [ ] Altre condizioni

11. **Uso di sigarette:** *
*Mark only one oval.*

- ( ) Ho fumato in passato
- ( ) Non fumo
- ( ) Fumo adesso

12. **Se hai risposto FUMO, indica quante sigarette al giorno:**

..............................................................................

13. **Consumo di alcool:** *
*Mark only one oval.*

- ( ) No
- ( ) Occasionalmente

14. **Se hai risposto OCCASIONALMENTE, indica quanti bicchieri al giorno:**

..............................................................................

15. **Elenca problemi relativi al consumo di alcool:**

..............................................................................
..............................................................................
..............................................................................
..............................................................................
..............................................................................

16. **Prescrizioni mediche:** *
*Mark only one oval.*

- ( ) No
- ( ) Si

17. **Se hai risposto SI, indica le prescrizioni mediche:**
(Nome o principio attivo del farmaco e dosaggio)

..............................................................................
..............................................................................
..............................................................................
..............................................................................
..............................................................................

18. **Fai uso di integratori? Se si indica quali.**

..........................................................................

..........................................................................

..........................................................................

..........................................................................

..........................................................................

## Livello di attività fisica

19. **Come valuti il tuo livello di attività fisica nell'ultimo anno?** *

*Mark only one oval.*

- ( ) BASSO: stare seduti, piegarsi, guidare, parlare, no esercizio programmato
- ( ) MEDIO: stare in piedi, camminare, piegarsi muoversi
- ( ) MODERATO: stare in piedi, camminare, piegarsi, muoversi, fare esercizio una volta a settimana
- ( ) ATTIVO: lavoro fisico leggero, salire le scale, fare esercizio 2 o 3 giorni a settimana
- ( ) MOLTO ATTIVO: lavoro fisico moderato, esercizio regolare 4 o più giorni a settimana

20. **Attività fisica o sport praticato:** *

*Check all that apply.*

- [ ] Niente
- [ ] Palestra
- [ ] Corsa
- [ ] Nuoto
- [ ] Ciclismo
- [ ] Basket
- [ ] Calcio
- [ ] Golf
- [ ] Tennis
- [ ] Atletica
- [ ] Other: ..........................................................................

21. **Quante volte ti alleni a settimana?** *

*Mark only one oval.*

- ( ) 0
- ( ) 1
- ( ) 2
- ( ) 3
- ( ) 4
- ( ) 5
- ( ) 6
- ( ) 7

22. **Quante ore ti alleni a settimana?** *

*Mark only one oval.*

- 0
- 1 - 2 ore
- 2 -3 ore
- 3 - 6 ore
- più di 6 ore

23. **Quanto dura mediamente una seduta di allenamento?** *

*Mark only one oval.*

- Non mi alleno
- meno di 1 ora
- 1 - 2 ore
- 2 - 3 ore
- più di 3 ore
- Other: ..........

24. **Hai un allenatore che ti segue?** *

*Mark only one oval.*

- Si
- No

25. **Che tipo di attività fisica svolgi?** *

*Mark only one oval.*

- Nessuna attività
- Ricreazionale/amatoriale
- Agonistica (partecipazione a campionati)

26. **Se hai risposto AGONISTICA alla domanda precedente, indica a quante gare partecipi mediamente ogni mese:**

..........

## BODY IMAGE DIMENSIONAL ASSESSMENT (BIDA)

In riferimento alla figura riportata, rispondi alle seguenti domande utilizzando la scala che oscilla tra i valori 1,8 e 5,2 in merito alla percezione della dimensione corporea. Naturalmente sono consentite risposte intermedie (es. 3,6).

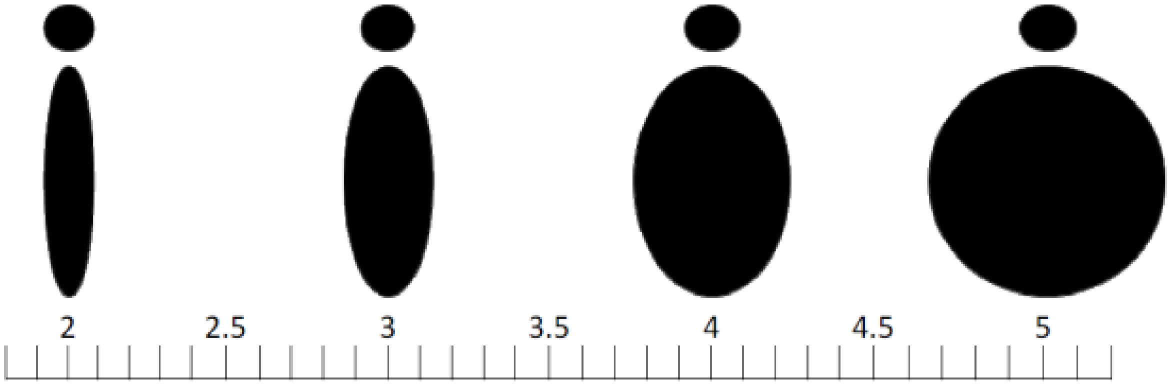


27. **Come pensi di essere attualmente?** *

28. **Quale è la tua figura ideale?** *

29. **Quale figura pensi sia più attraente per l'altro sesso?** *

30. **Come sono la maggior parte delle persone del tuo sesso ed età?** *

31. **Indica il tuo peso** *

32. **Indica la tua statura** *

# EATING ATTITUDES TEST (EAT-26)

Questo questionario permette di determinare la possibilità di avere un disturbo alimentare che necessità di attenzione da parte di un medico. Questo questionario non è progettato per fare una diagnosi di un disturbo alimentare o di prendere il posto di una consulenza medica professionale.

33. **Peso attuale** *

34. **Peso maggiore** *
(gravidanza esclusa)

35. **Peso minore** *
(durante l'età adulta - età superiore ai 25 anni)

36. **Peso ideale** *

.......................................................................................................................

37. **1. Ho una terribile paura di ingrassare** *

*Mark only one oval.*

- Sempre
- Molto spesso
- Spesso
- Qualche volta
- Raramente
- Mai

38. **2. Quando ho fame evito di mangiare** *

*Mark only one oval.*

- Sempre
- Molto spesso
- Spesso
- Qualche volta
- Raramente
- Mai

39. **3. Penso al cibo con preoccupazione** *

*Mark only one oval.*

- Sempre
- Molto spesso
- Spesso
- Qualche volta
- Raramente
- Mai

40. **4. Mi è capitato di mangiare con enorme voracità sentendomi incapace di smettere** *

*Mark only one oval.*

- Sempre
- Molto spesso
- Spesso
- Qualche volta
- Raramente
- Mai

41. **5. Ho l'abitudine di sminuzzare il cibo** *
*Mark only one oval.*

- Sempre
- Molto spesso
- Spesso
- Qualche volta
- Raramente
- Mai

42. **6. Faccio molta attenzione al potere calorico dei cibi che mangio** *
*Mark only one oval.*

- Sempre
- Molto spesso
- Spesso
- Qualche volta
- Raramente
- Mai

43. **7. Evito in particolare i cibi con elevato contenuto di carboidrati (pane, pasta, dolci)** *
*Mark only one oval.*

- Sempre
- Molto spesso
- Spesso
- Qualche volta
- Raramente
- Mai

44. **8. Sento che gli altri vorrebbero che io mangiassi di più** *
*Mark only one oval.*

- Sempre
- Molto spesso
- Spesso
- Qualche volta
- Raramente
- Mai

45. **9. Mi capita di vomitare dopo aver mangiato** *
*Mark only one oval.*

- Sempre
- Molto spesso
- Spesso
- Qualche volta
- Raramente
- Mai

46. **10. Mi sento molto in colpa dopo mangiato** *

*Mark only one oval.*

- Sempre
- Molto spesso
- Spesso
- Qualche volta
- Raramente
- Mai

47. **11. Mi tormenta il desiderio di essere più magro/a** *

*Mark only one oval.*

- Sempre
- Molto spesso
- Spesso
- Qualche volta
- Raramente
- Mai

48. **12. Mi sottopongo a esercizi fisici intensi per bruciare calorie** *

*Mark only one oval.*

- Sempre
- Molto spesso
- Spesso
- Qualche volta
- Raramente
- Mai

49. **13. Gli altri pensano che sono troppo magro/a** *

*Mark only one oval.*

- Sempre
- Molto spesso
- Spesso
- Qualche volta
- Raramente
- Mai

50. **14. Mi preoccupa l'idea di avere del grasso sul corpo** *

*Mark only one oval.*

- Sempre
- Molto spesso
- Spesso
- Qualche volta
- Raramente
- Mai

51. **15. Impiego più tempo degli altri per mangiare** *

*Mark only one oval.*

- Sempre
- Molto spesso
- Spesso
- Qualche volta
- Raramente
- Mai

52. **16. Evito cibi dolci** *

*Mark only one oval.*

- Sempre
- Molto spesso
- Spesso
- Qualche volta
- Raramente
- Mai

53. **17. Mangio cibi dietetici** *

*Mark only one oval.*

- Sempre
- Molto spesso
- Spesso
- Qualche volta
- Raramente
- Mai

54. **18. Sento che il cibo domina la mia vita** *

*Mark only one oval.*

- Sempre
- Molto spesso
- Spesso
- Qualche volta
- Raramente
- Mai

55. **19. Mi piace mostrare un grande autocontrollo verso il cibo e dominare la fame** *

*Mark only one oval.*

- Sempre
- Molto spesso
- Spesso
- Qualche volta
- Raramente
- Mai

56. **20. Sento che gli altri fanno pressioni su di me perché io mangi** *

*Mark only one oval.*

- Sempre
- Molto spesso
- Spesso
- Qualche volta
- Raramente
- Mai

57. **21. Dedico al cibo troppo tempo e troppi pensieri** *

*Mark only one oval.*

- Sempre
- Molto spesso
- Spesso
- Qualche volta
- Raramente
- Mai

58. **22. Mi dispero se mangio dei dolci** *

*Mark only one oval.*

- Sempre
- Molto spesso
- Spesso
- Qualche volta
- Raramente
- Mai

59. **23. Mi impegno in programmi di dieta** *

*Mark only one oval.*

- Sempre
- Molto spesso
- Spesso
- Qualche volta
- Raramente
- Mai

60. **24. Mi piace che il mio stomaco sia vuoto** *

*Mark only one oval.*

- Sempre
- Molto spesso
- Spesso
- Qualche volta
- Raramente
- Mai

61. **25. Ho l'impulso di vomitare dopo mangiato** *

*Mark only one oval.*

- Sempre
- Molto spesso
- Spesso
- Qualche volta
- Raramente
- Mai

62. **26. Mi piace provare nuovi cibi elaborati** *

*Mark only one oval.*

- Sempre
- Molto spesso
- Spesso
- Qualche volta
- Raramente
- Mai

# EXERCISE DEPENDENCE SCALE-21

Usando una scala da 1 (mai) a 6 (sempre), si prega di completare le seguenti domande nel modo più onesto possibile. Le domande si riferiscono ai pensieri ed i comportamenti avuti negli ultimi 3 mesi riguardanti l'esercizio fisico. Non ci sono risposte giuste o sbagliate. Tutte le risposte sono confidenziali.

63. **1. Mi esercito per evitare di essere irritabile** *

*Mark only one oval.*

| | 1 | 2 | 3 | 4 | 5 | 6 | |
|---|---|---|---|---|---|---|---|
| Mai | | | | | | | Sempre |

64. **2. Mi esercito nonostante abbia problemi fisici ricorrenti** *

*Mark only one oval.*

| | 1 | 2 | 3 | 4 | 5 | 6 | |
|---|---|---|---|---|---|---|---|
| Mai | | | | | | | Sempre |

65. **3. Aumento costantemente l'intensità della mia attività fisica per raggiungere gli effetti/benefici desiderati** *

*Mark only one oval.*

| | 1 | 2 | 3 | 4 | 5 | 6 | |
|---|---|---|---|---|---|---|---|
| Mai | | | | | | | Sempre |

66. **4. Non riesco a ridurre la durata della mia attività fisica** *

*Mark only one oval.*

| | 1 | 2 | 3 | 4 | 5 | 6 | |
|---|---|---|---|---|---|---|---|
| Mai | | | | | | | Sempre |

67. **5. Vorrei esercitarmi piuttosto che trascorrere del tempo con la famiglia/gli amici** *

*Mark only one oval.*

| | 1 | 2 | 3 | 4 | 5 | 6 | |
|---|---|---|---|---|---|---|---|
| Mai | | | | | | | Sempre |

68. **6. Passo molto tempo facendo attività fisica** *

*Mark only one oval.*

| | 1 | 2 | 3 | 4 | 5 | 6 | |
|---|---|---|---|---|---|---|---|
| Mai | | | | | | | Sempre |

69. **7. Faccio attività fisica più a lungo di quanto voglio** *

*Mark only one oval.*

| | 1 | 2 | 3 | 4 | 5 | 6 | |
|---|---|---|---|---|---|---|---|
| Mai | | | | | | | Sempre |

70. **8. Mi esercito per evitare di sentirmi ansioso** *

*Mark only one oval.*

| | 1 | 2 | 3 | 4 | 5 | 6 | |
|---|---|---|---|---|---|---|---|
| Mai | | | | | | | Sempre |

71. **9. Mi esercito quando sono infortunato** *

*Mark only one oval.*

| | 1 | 2 | 3 | 4 | 5 | 6 | |
|---|---|---|---|---|---|---|---|
| Mai | | | | | | | Sempre |

72. **10. Incremento costantemente la frequenza della mia attività fisica per raggiungere gli effetti/benefici desiderati** *

*Mark only one oval.*

| | 1 | 2 | 3 | 4 | 5 | 6 | |
|---|---|---|---|---|---|---|---|
| Mai | | | | | | | Sempre |

73. **11. Sono incapace di ridurre la frequenza della mia attività fisica** *

*Mark only one oval.*

| | 1 | 2 | 3 | 4 | 5 | 6 | |
|---|---|---|---|---|---|---|---|
| Mai | | | | | | | Sempre |

74. **12. Penso all’esercizio fisico quando dovrei essere concentrato sulla scuola/sul lavoro** *

*Mark only one oval.*

| | 1 | 2 | 3 | 4 | 5 | 6 | |
|---|---|---|---|---|---|---|---|
| Mai | | | | | | | Sempre |

75. **13. Passo la maggior parte del mio tempo libero facendo esercizio fisico** *

*Mark only one oval.*

| | 1 | 2 | 3 | 4 | 5 | 6 | |
|---|---|---|---|---|---|---|---|
| Mai | | | | | | | Sempre |

76. **14. Mi esercito di più di quanto aspettavo** *

*Mark only one oval.*

| | 1 | 2 | 3 | 4 | 5 | 6 | |
|---|---|---|---|---|---|---|---|
| Mai | | | | | | | Sempre |

77. **15. Mi esercito per evitare di sentirmi teso** *

*Mark only one oval.*

| | 1 | 2 | 3 | 4 | 5 | 6 | |
|---|---|---|---|---|---|---|---|
| Mai | | | | | | | Sempre |

78. **16. Mi esercito nonostante i miei persistenti problemi fisici** *

*Mark only one oval.*

| | 1 | 2 | 3 | 4 | 5 | 6 | |
|---|---|---|---|---|---|---|---|
| Mai | | | | | | | Sempre |

79. **17. Aumento costantemente la durata della mia attività fisica per raggiungere gli effetti/benefici desiderati** *

*Mark only one oval.*

| | 1 | 2 | 3 | 4 | 5 | 6 | |
|---|---|---|---|---|---|---|---|
| Mai | | | | | | | Sempre |

80. **18. Sono incapace di ridurre l'intensità della mia attività fisica** *
*Mark only one oval.*

| | 1 | 2 | 3 | 4 | 5 | 6 | |
|---|---|---|---|---|---|---|---|
| Mai | ◯ | ◯ | ◯ | ◯ | ◯ | ◯ | Sempre |

81. **19. Scelgo di esercitarmi per evitare di perdere tempo con famiglia/amici** *
*Mark only one oval.*

| | 1 | 2 | 3 | 4 | 5 | 6 | |
|---|---|---|---|---|---|---|---|
| Mai | ◯ | ◯ | ◯ | ◯ | ◯ | ◯ | Sempre |

82. **20. Una buona parte del mio tempo la passo ad esercitarmi** *
*Mark only one oval.*

| | 1 | 2 | 3 | 4 | 5 | 6 | |
|---|---|---|---|---|---|---|---|
| Mai | ◯ | ◯ | ◯ | ◯ | ◯ | ◯ | Sempre |

83. **21. Mi esercito più di quanto pianifico** *
*Mark only one oval.*

| | 1 | 2 | 3 | 4 | 5 | 6 | |
|---|---|---|---|---|---|---|---|
| Mai | ◯ | ◯ | ◯ | ◯ | ◯ | ◯ | Sempre |

Powered by
Google Forms
